# High Instantaneous Inhibitory Potential of Bictegravir and the New Spiro-β-Lactam BSS-730A for HIV-2 Isolates from RAL-Naïve and RAL-Failing Patients

**DOI:** 10.3390/ijms232214300

**Published:** 2022-11-18

**Authors:** Inês Bártolo, Inês Moranguinho, Paloma Gonçalves, Ana Rita Diniz, Pedro Borrego, Francisco Martin, Inês Figueiredo, Perpétua Gomes, Fátima Gonçalves, Américo J. S. Alves, Nuno Alves, Umbelina Caixas, Inês V. Pinto, Isabel Barahona, Teresa M. V. D. Pinho e Melo, Nuno Taveira

**Affiliations:** 1 Instituto de Investigação do Medicamento (iMed.ULisboa), Faculdade de Farmácia, Universidade de Lisboa, 1649-019 Lisboa, Portugal; 2 Centro de Investigação Interdisciplinar Egas Moniz (CiiEM), Instituto Superior de Ciências da Saúde Egas Moniz, 2829-511 Caparica, Portugal; 3 Centro de Administração e Políticas Públicas (CAPP), Instituto Superior de Ciências Sociais e Políticas (ISCSP), Universidade de Lisboa, 1649-019 Lisboa, Portugal; 4 Laboratório de Biologia Molecular, LMCBM, SPC, Centro Hospitalar Lisboa Ocidental–HEM, 1649-019 Lisboa, Portugal; 5 Department of Chemistry, Coimbra Chemistry Centre-Institute of Molecular Sciences (CQC-IMS), University of Coimbra, 3004-535 Coimbra, Portugal; 6 Serviço de Medicina 1.4, Hospital de S. José, CHLC, EPE, and Faculdade de Ciências Médicas, FCM-Nova, Centro de Estudos de Doenças Crónicas–CEDOC, 1649-019 Lisboa, Portugal; 7 Medicina Interna, Hospital de Cascais Dr. José de Almeida, 2755-009 Alcabideche, Portugal

**Keywords:** HIV-2, antiretroviral activity, integrase inhibitors (INIs), spiro-β-lactam BSS-730A, instantaneous inhibitory potential (IIP), drug resistance

## Abstract

Integrase inhibitors (INIs) are an important class of drugs for treating HIV-2 infection, given the limited number of drugs active against this virus. While the clinical efficacy of raltegravir and dolutegravir is well established, the clinical efficacy of bictegravir for treating HIV-2 infected patients has not been determined. Little information is available regarding the activity of bictegravir against HIV-2 isolates from patients failing raltegravir-based therapy. In this study, we examined the phenotypic and matched genotypic susceptibility of HIV-2 primary isolates from raltegravir-naïve and raltegravir-failing patients to raltegravir, dolutegravir, and bictegravir, and to the new spiro-β-lactam BSS-730A. The instantaneous inhibitory potential (IIP) was calculated to help predict the clinical activity of bictegravir and BSS-730A. Isolates from raltegravir-naïve patients were highly sensitive to all INIs and BSS-730A. Combined integrase mutations E92A and Q148K conferred high-level resistance to raltegravir, and E92Q and T97A conferred resistance to raltegravir and dolutegravir. The antiviral activity of bictegravir and BSS-730A was not affected by these mutations. BSS-730A displayed strong antiviral synergism with raltegravir. Mean IIP values at Cmax were similar for all INIs and were not significantly affected by resistance mutations. IIP values were significantly higher for BSS-730A than for INIs. The high IIP values of bictegravir and BSS-730A for raltegravir-naïve and raltegravir-resistant HIV-2 isolates highlight their potential value for treating HIV-2 infection. Overall, the results are consistent with the high clinical efficacy of raltegravir and dolutegravir for HIV-2 infection and suggest a promising clinical profile for bictegravir and BSS-730A.

## 1. Introduction

Human immunodeficiency virus type 2 (HIV-2) originated in West Africa around 1938 [1] and was isolated from Guinea-Bissau and Cape Verde patients with AIDS in 1986 [2]. These countries, along with Senegal and Côte d’Ivoire, were the main sources of extra-regional viral migration. Portugal and France, the two first targets of extra-regional viral dispersion from West Africa [3], report a relatively high percentage of cases of HIV-2 infection (3.3% [4] and 1.8% [5] of all HIV cases, respectively). HIV-2 has spread globally, and current estimates indicate that approximately 1 to 2 million people worldwide live with HIV-2 [6]. HIV-2 is composed of nine groups termed A to I, of which group A is by far the most disseminated [7]. HIV-2 is an interesting model of attenuated HIV disease. It is less pathogenic than HIV-1, more than doubling the average time from asymptomatic infection to AIDS and death in untreated patients [8,9,10,11]. Nonetheless, without effective antiretroviral therapy (ART), most infected individuals will progress to AIDS and die [11,12].

Due to major sequence and structural differences in the *pol* and *env* genes [13,14,15,16], HIV-2 is naturally resistant to many of the drugs in clinical use for HIV-1 infected patients. Hence, HIV-2 is naturally resistant to non-nucleoside reverse transcriptase inhibitors [17], to the fusion inhibitor enfuvirtide [18,19,20], to the attachment-inhibitor temsavir [21], and to broadly neutralizing monoclonal antibodies used for prevention and therapy of HIV infection [22,23,24]. Moreover, HIV-2 has reduced sensitivity to the protease inhibitors amprenavir, nelfinavir, ritonavir, indinavir, atazanavir, and tipranavir [16,17,25,26,27,28,29,30]. Additional constraints to the effective treatment of HIV-2 infected patients are the poor CD4^+^ T cell recovery following ART [31,32] and the low genetic barrier to resistance of HIV-2 [29,33]. Hence, antiretroviral therapy for HIV-2 patients should be well planned and managed to assure long-term efficacy and prevent the development of resistance.

First-generation integrase inhibitors (INI), raltegravir (RAL) and elvitegravir (EVG), inhibit HIV-2 replication in vitro with an IC_50_ range similar to that of HIV-1 [34] and reduce viral load to undetectable levels in HIV-2 infected patients when combined with other suppressive antiretroviral drugs [35,36,37,38]. Significant cross-resistance between RAL and EVG prevents sequential therapy with these drugs for both HIV-1 and HIV-2 infected patients [39,40,41,42]. Second-generation INIs, dolutegravir (DTG), bictegravir (BIC), and cabotegravir (CAB), have been less studied but also seem to be potent inhibitors of HIV-2 isolates obtained from INI-naïve patients with an IC_50_ range similar to that of HIV-1 (nM or sub-nM IC_50_ or EC_50_ range) [37,43,44,45,46,47].

The clinical effectiveness of DTG has been examined in a few HIV-2 infected individuals failing RAL-based regimens with some different results. In two studies, virus replication was fully suppressed by DTG in patients failing RAL due to the N155H mutation [36,37]. In another study, DTG-containing regimens were not effective in patients harboring RAL-resistant viruses with mutations Q148H/R/K and N155H [48]. A 7 to 18-fold increase in DTG EC_50_ values was observed in isolates obtained from two RAL-experienced patients with double (T97A + Y143C; G140S + Q148R) and triple (G140T + Q148R + N155H) resistance mutations [43]. Other mutations that may contribute to DTG resistance in the clinical setting are G118R [36] and R263K + E92G [49].

There are no studies examining the clinical effectiveness of BIC in HIV-2 infected individuals, and the two studies that have examined the susceptibility of INI-resistant isolates to BIC report conflicting results. In one study, BIC inhibited the replication of some HIV-2 isolates bearing major resistance mutations at codons 143, 148, and 155 and insertions at codon 231 [47]. On the other hand, isolates with the G140S/Q148R and G140S/Q148H mutations were 34- and 110-fold resistant to BIC, respectively; other resistance-associated mutations conferred less than 5-fold changes in BIC susceptibility [46]. A consensus has been reached recently by an HIV-2 EU expert group [50], according to which high-level HIV-2 resistance to BIC is associated with mutations G140S + Q148H/R, Q148K, E92Q + N155H, and T97A + N155H and intermediate level resistance with mutations E92Q, Q148H/R, N155H, T97A + Y143C, and 231INS. Overall, these studies reveal a remarkable heterogeneity in RAL, DTG, and BIC resistance mutations and resistance pathways in HIV-2, making the prediction of genetic susceptibility to these drugs a major challenge [51].

The scarcity of drugs active against HIV-2 and the increasing resistance of HIV-1 and HIV-2 to INIs clearly indicates the need for additional drugs that are effective against both viruses [52,53]. Our previous research on spiro-β-lactams led to the discovery of several compounds with remarkable anti-HIV-1 activity [54,55,56]. The lead compound, BSS-730A, acts in synergy with AMD3100, an HIV entry inhibitor, against the HIV-1 SG3.1 strain and has a high selectivity index in a human cell line (CC_50_/EC_50_ > 2946), emerging as an excellent candidate for further development as an anti-HIV drug [56]. The mechanism of action of BSS-730A remains to be determined.

In this study, we examined the phenotypic and matched genotypic susceptibility of a large panel of primary isolates obtained from RAL-naïve and RAL-experienced HIV-2 infected patients from Portugal to RAL, DTG, BIC, and BSS-730A. BIC and BSS-730A have never been used in HIV-2 infected patients. To better understand the clinical profile of these drugs in HIV-2 infected patients, we calculated their instantaneous inhibitory potential (IIP) in a single-round infectivity assay.

## 2. Results

### 2.1. Phenotypic Susceptibility of Viral Isolates to INIs and BSS-730A

**Figure 1:**
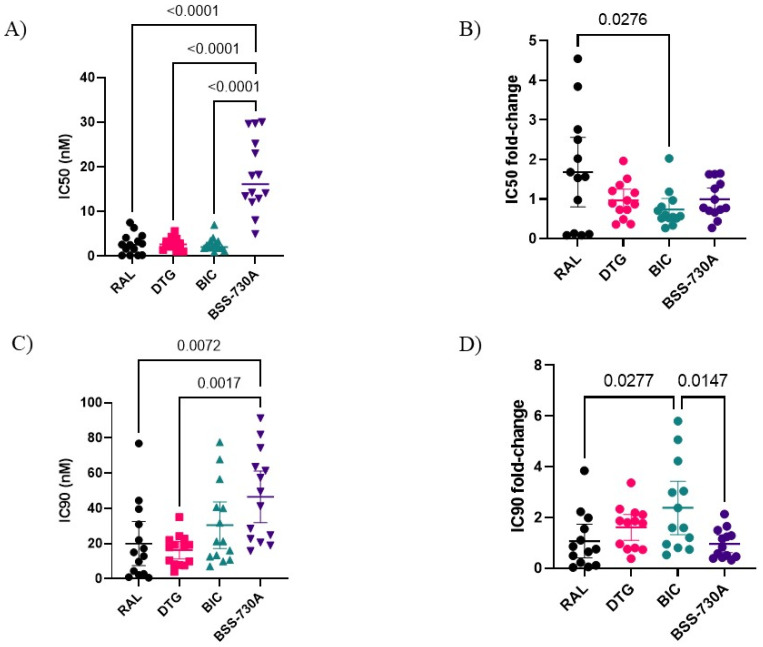
Susceptibility of isolates from RAL-naïve patients to the four drugs. (**A**) Comparison of mean IC_50_ (nM) values of all drugs; (**B**) Comparison of IC_50_ fold-change; (**C**) Comparison of mean IC_90_ (nM) values; (**D**) Comparison of IC_90_ fold-change. One-way ANOVA with Tukey’s multiple comparisons test was used. Only *p*-values < 0.05 are shown. Lines indicate mean values and 95% CI.

The sensitivity of the isolates from RAL-naïve patients to the four drugs differed significantly (IC_50_, F = 43.38; *p* < 0.0001; IC_90_, F = 5.99; *p* = 0.0014). In general, isolates were easier to inhibit with BIC (IC_50_) or DTG (IC_90_) than with BSS-730A (Table 1, Supplementary Tables S1 and S2; Figure 1). Isolates 03PTHCC1 and 03PTHDECT presented, respectively, a 3.8-fold and 4.5-fold increase in RAL IC_50_ relative to HIV-2 ROD (Supplementary Table S1) despite having no resistance mutations to RAL.

Isolates from RAL-experienced patients were 29.04-fold less sensitive to RAL than isolates from naïve patients (mean IC_50_ of isolates from RAL-experienced patients 78.178 nM vs. 2.692 nM, *p* = 0.0294) (Table 1 and Supplementary Table S1). Patient Fourteen’s isolate (15PTHCEC) showed high-level resistance to RAL, whereas Patient Ten’s isolate from 2010 (10PTHSJIG) showed intermediate-level resistance. Interestingly, in 2015, when this patient was undergoing a relatively successful DTG-based regimen (Supplementary Figure S1; Supplementary Table S3), the virus isolate had no resistance mutations and was fully sensitive to RAL, indicating a full DTG-driven replacement of the virus quasispecies in the peripheral blood of this patient.

DTG was highly active against all isolates from RAL-naïve patients and against two out of three isolates from RAL-experienced patients (Supplementary Tables S1 and S2). Of note, isolate 10PTHSJIG showed intermediate-level resistance to DTG, increasing 5.2-fold the mean IC_50_ of DTG for isolates from RAL-experienced patients relative to isolates from naïve patients (mean IC_50_, 14.467 nM vs. 2.778 nM; *p* = 0.0059) (Table 1 and Supplementary Table S1).

BIC and BSS-730A were the only drugs that presented similar activity against isolates from RAL-naïve and RAL-experienced patients (Table 1, Supplementary Tables S1 and S2). Nonetheless, the mean IC_90_ of BIC for isolates from RAL-experienced patients was 3.6-fold higher relative to BSS-730A (Table 1 and Supplementary Table S2). Similar results were obtained when comparing the IC_90_ of BSS-730A with DTG (2.1-fold difference) and RAL (5.5-fold difference). These results confirm BIC as a potent inhibitor of HIV-2 and suggest that BSS-730A could be highly effective at treating infections caused by RAL-resistant HIV-2 strains.

### 2.2. Curve Slope and Instantaneous Inhibitory Potential (IIP)

**Figure 2:**
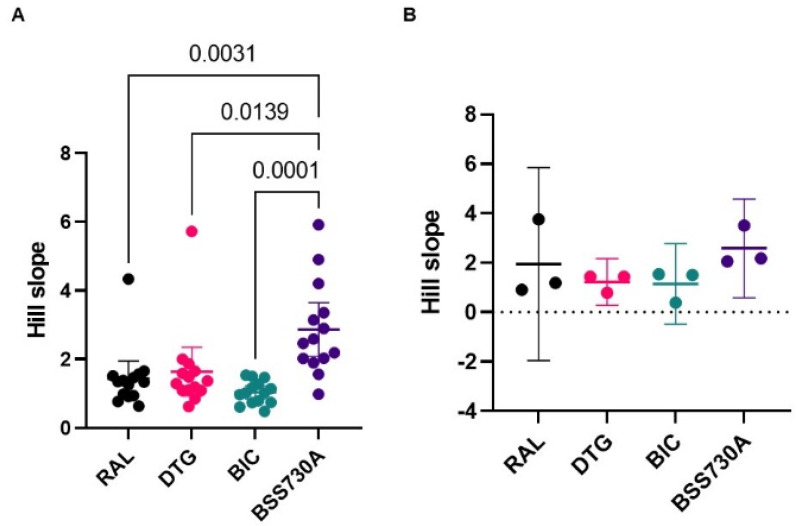
Hill slope values of RAL, DTG, BIC, and BSS-730A for HIV-2 isolates. (**A**) Isolates from RAL-naïve patients; (**B**) isolates from RAL-experienced patients. Lines indicate mean values with 95% CI. One-way ANOVA with Tukey’s multiple comparisons test was used. Only *p*-values < 0.05 are shown.

IIP provides a more accurate measure of antiviral activity than IC_50_ as it also considers the slope of the dose-response curve and clinically relevant drug concentrations (Cmax or Cmin). In this study, mean slope values were higher for BSS-730A and lower for BIC, especially for isolates from RAL-naïve patients (Figure 2, Supplementary Table S4). There was no significant difference in mean slope values obtained with isolates from RAL-naïve and RAL-experienced patients for any of the drugs (Table 1).

**Figure 3:**
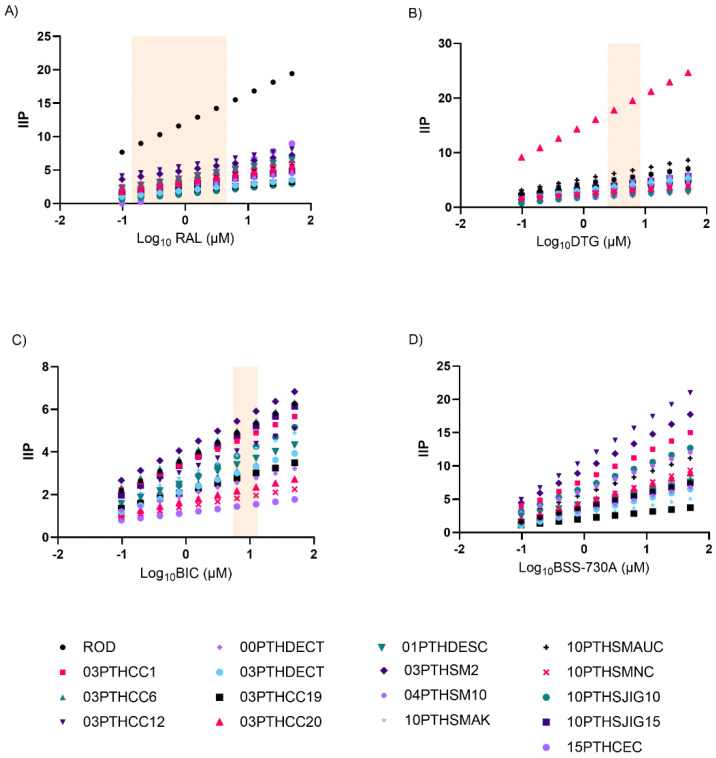
IIP values of RAL (**A**), DTG (**B**), BIC (**C**), and BSS-730A (**D**) for HIV-2 isolates at different concentration ranges. The rectangle limits the range of clinical concentrations, Cmax and Cmin, determined for each INI. Cmax and Cmin have not yet been defined for BSS-730A.

IIP values for all isolates at the different drug concentration ranges are shown in Figure 3 and Supplementary Table S5. The overall high IIP values of RAL for HIV-2ROD and of DTG for isolate 03PTHCC20 despite within average IC_50_ values (1.649 nM of RAL and 2.478 nM for DTG) were related to the unusually high slope values for these viruses, 4.337 and 5.725, respectively. Thus, infections caused by these viruses should be more responsive to treatment with these drugs than expected by considering the IC_50_ alone.

**Figure 4:**
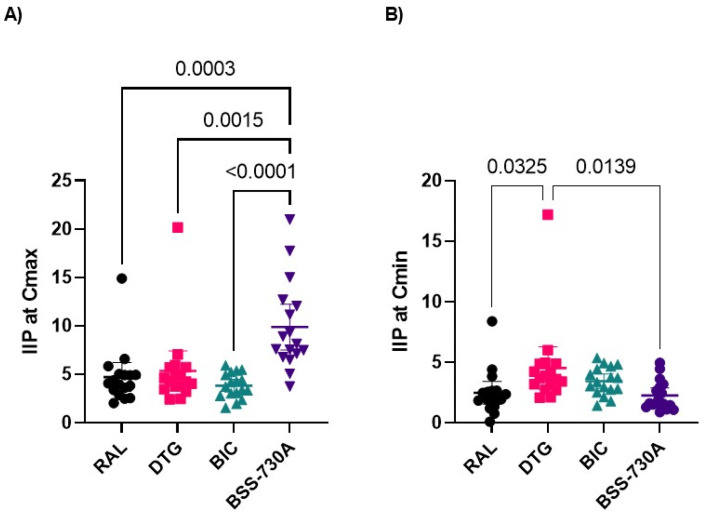
Comparison of IIP values of RAL, DTG, BIC, and BSS-730A at Cmax (**A**) and Cmin (**B**). Lines indicate mean values with 95% CI. One-way ANOVA with Tukey’s multiple comparisons test was used. Only *p*-values < 0.05 are shown.

Mean IIP values of BSS-730A at Cmax were significantly higher than RAL, DTG, and BIC (Figure 4; Supplementary Table S5). IIP values were similar for all INIs at Cmax and higher for DTG at Cmin.

**Figure 5:**
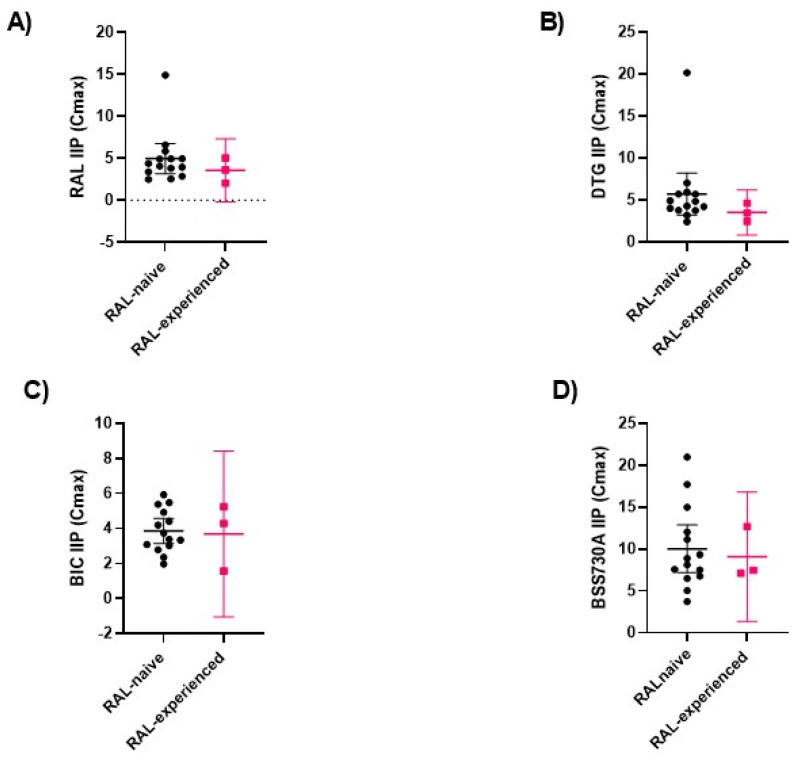
Comparison of IIP values of RAL (**A**), DTG (**B**), BIC (**C**), and BSS-730A (**D**) at Cmax for isolates from RAL-naïve and RAL-experienced patients. The Mann–Whitney test was used. Lines indicate mean values with 95% CI. Only *p*-values < 0.05 are shown.

RAL-resistance mutations modestly reduced mean IIP values for all INIs and BSS-730A. IIP reduction was higher for DTG (1.63-fold) and RAL (1.40-fold) and lower for BSS-730A (1.10-fold) and BIC (1.04-fold), suggesting that at clinically relevant concentrations, BSS-730A and BIC will be less affected by RAL-resistance mutations (Figure 5; Supplementary Table S5).

### 2.3. Antiviral Activity of BSS-730A and RAL Are Synergic

The activity of BSS-730A was assessed in combination with RAL in a single-cycle infectivity assay with the primary isolate 03PTHCC19. The assay was performed with 1:1, 1:3, and 3:1 RAL: BSS-730A ratios. Combination indices (CI) were calculated to determine whether synergistic, additive, or antagonistic effects occurred in these combinations. There was a strong synergism at all virus inhibition levels when BSS-730A was added in equal or higher concentration than RAL (CI: 0.168–0.243) (Table 2). When RAL was used in higher concentrations, a very strong synergism was observed (CI: 0.052–0.094). These results suggest that BSS-730A could be used in combination with RAL and likely other integrase inhibitors to treat HIV-2 infection.

### 2.4. Analysis of Genotypic Drug Resistance

Integrase sequences were produced from all isolates, and their evolutionary relationships, as well as polymorphisms and resistance mutations, were investigated. In phylogenetic analysis, all new isolates belonged to group A which is the most common HIV-2 group in Portugal and worldwide (Supplementary Figure S2) [7]. As expected, the sequences from the two isolates from patient 10 (10PTHJSIG and 15PTHJSIG) formed a monophyletic cluster supported by high bootstrap values. Likewise, the sequences from isolates of Patient 1 (00PTHDECT and 03PTHDECT) formed a strongly supported cluster with the sequence from Patient 7 isolate (03PTHCC20), confirming the common ancestry of the isolates and the epidemiologic link between these patients (child and mother) [57].

Isolates from RAL-naïve patients lacked drug resistance mutations. As for isolates from RAL-experienced patients, the 2010 isolate of Patient 10 (10PTHSJIG) had the major resistance mutation E92Q and the accessory mutation T97A (Table 3). Consistent with our phenotypic results, E92Q, in combination with T97A, has been found to reduce susceptibility to DTG [50,58] and, in combination with N155H and T97A/Y143C, to cause major resistance to RAL [42,43,44,59]. The 2015 isolate from Patient 10 (15PTHSJIG) had no major resistance mutations but harbored I84V, a polymorphism that is significantly more common in INI-experienced than in INI-naïve persons [58]. Finally, isolate from Patient 14 (15PTHCEC) had the major resistance mutations E92A and Q148K and the accessory mutation I84V (Table 3). Q148K has been associated with reduced susceptibility to each of the INIs [58].

## 3. Discussion

We showed that integrase inhibitors RAL, DTG, BIC, and the spiro-β-lactam BSS-730A were potent inhibitors of group A primary HIV-2 isolates from RAL-naïve patients. However, the sensitivity of these wild-type isolates to the four drugs differed significantly, and, in general, isolates were easier to inhibit using BIC or DTG than by RAL or BSS-730A. Two of the three isolates from RAL-experienced patients were resistant to RAL, and one (10PTHSJIG) also showed intermediate-level resistance to DTG. In contrast, BIC, and especially BSS-730A, presented similar activity against isolates from RAL-naïve and RAL-experienced patients. We also showed that BSS-730A had a very strong synergistic effect when combined with RAL. Together, these results confirm and extend previous data on the anti-HIV-2 activity of DTG [36,37,43,44,48] and BIC [46,47] and demonstrate that BSS-730A is a potent inhibitor of HIV-2 that may be useful in treating infections caused by HIV-2 either alone or in combination with INIs.

Two RAL-resistant isolates had resistance-associated mutations in the integrase already described in the literature. Isolate 10PTHSJIG from Patient 10, which is resistant to RAL and showed intermediate-level resistance to DTG, carried the major mutation E92Q and secondary mutation T97A. Our phenotypic results are consistent with previous studies showing that E92Q, alone or in combination with N155H and T97A/Y143C, confers HIV-2 intermediate- to high-level resistance to RAL [42,43,44,51,59,60] and, in combination with T97A, confers intermediate resistance to DTG [50,51,60]. The 2015 isolate from this patient (15PTHSJIG) was fully sensitive to all INIs, had no resistance mutations, and harbored I84V, a polymorphism that is unrelated to drug resistance [60] but is significantly more common in INI-experienced than in INI-naïve persons [51].

Isolate 15PTHCEC carried the E92A and Q148K mutations which were associated with high-level resistance to RAL and a minor decrease (2.032-fold) in sensitivity to DTG relative to isolates from RAL-naïve patients. To our knowledge, this combination of mutations has never been described in HIV-1 and HIV-2 infected patients. Q148K confers high-level resistance to RAL in HIV-2, especially when in association with other mutations (e.g., E92Q, T97A, G140S) [34,48,51,60,61,62,63]. Clinical studies have shown that HIV-2 patients failing DTG after RAL resistance due to N155H often select mutations at codon 148 (Q148K or Q148R) [36,48]. This usually occurs in combination with accessory mutations at codons 151 and 153 and different polymorphisms. In vitro studies with site-directed mutants have also shown that Q148K alone moderately affects the susceptibility of reference isolate HIV-2ROD9 to DTG [44,60]. As for the E92A secondary mutation in HIV-2 ROD9, this mutation alone conferred low-level resistance to DTG and low- to intermediate-level resistance to RAL and DTG when in combination with other mutations such as T97A and N155H [60]. Overall, our study highlights the added value of phenotypic assays to assess HIV-2 susceptibility to INIs and contributes to producing better genotypic algorithms for predicting HIV-2 susceptibility to these antiretroviral drugs.

To better understand the clinical profile of these drugs in HIV-2 infected patients, we determined their IIP values. IIP provides a more accurate measure of antiviral activity than IC_50_ and, in general, correlates better with clinical outcomes because it also considers the slope of the dose-response curve and clinically relevant drug concentrations [64,65]. IC_50_ alone also tends to underestimate the degree of resistance [66]. In this study, the first to address this issue in HIV-2, mean IIP values at Cmax were similar for all INIs and were not significantly affected by resistance mutations. Importantly, IIP values were 3-to-5 fold higher than those found for HIV-1 [64]. These results confirm the high efficacy of RAL and DTG in the treatment of HIV-2 infection [35,36,37,38] and suggest that BIC should also be highly effective at treating HIV-2 infection. Additional studies with more resistant viruses are needed to examine the impact of resistance mutations on the IIP of INIs in HIV-2. Strikingly, despite the lower IC_50_, slope and IIP values were significantly higher for BSS-730A than for INIs, further highlighting the clinical potential of this compound.

## 4. Materials and Methods

### 4.1. Ethics

Ethical approval for this study was obtained from the Ethics Committee of Hospital de S. José (DC-5125911). All patients provided informed consent prior to the start of the study. This research complies with the Declaration of Helsinki and the Oviedo Bioethics Convention on medical research in humans.

### 4.2. Cells, Plasmids, and Drugs

HEK-293T cells were purchased from American Type Culture Collection (Rockville, MD). TZM-bl cells and RAL were provided by the AIDS Research and Reference Reagent Program, National Institutes of Health. DTG and BIC were acquired from Quimigen, Portugal (https://www.quimigen.pt/ accessed on 1 July 2022). HEK-293T and TZM-bl cells were cultured in complete growth medium that consisted of Dulbecco’s minimal essential medium (DMEM) supplemented with 10% of fetal bovine serum, 100 U/mL of penicillin-streptomycin, 2 mM of L-Glutamine, 1 mM sodium pyruvate and 1x of MEM non-essential amino acids (Gibco/Invitrogen, Waltham, MA, USA). All cell cultures were maintained at 37 °C in 5% of CO_2_.

### 4.3. Patient Data

A total of sixteen primary isolates were obtained from fourteen HIV-2 infected Portuguese patients (Supplementary Table S3). Thirteen isolates originated from twelve INI-naïve patients and three from two RAL- experienced patients (10PTHSJIG, 15PTHSJIG, and 15PTHCEC). RAL-experienced Patient 10 provided two isolates, one at the end of May 2010 (10PTHSJIG) and the other in mid-June 2015 (15PTHSJIG). From 2007 to September 2010, this patient was given Combivir (AZT/3TC) + DRV/r + RAL (Supplementary Figure S1A). The first evidence of virologic failure was obtained in May 2009. In October 2010, a regimen with TDF + DRV/r + MVC150 was introduced with good results (undetectable viral load) until at least September 2011. From May to mid-August 2012, the patient interrupted the therapeutic due to travel to Guinea-Bissau; the patient resumed therapy on return, but virologic failure emerged due to low adherence. From October 2013 to August 2015, the patient was on 3TC alone. From August 12, 2015 onward, the patient was on DRV/r + MVC150 + DTG with continuous evidence of virologic suppression.

Isolate 15PTHCEC was obtained on 23 March 2015 from Patient 14, who was diagnosed with HIV-2 infection in 2010. Initial therapy in 2012 was DRV/r + FTC/TDF (Supplementary Figure S1B). Genotypic testing at the end of August 2013 showed resistance to all NRTIs and PIs used for HIV-2, and therapy was changed to MVC + RAL+ SQV/r with limited success, likely because the virus was already CXCR4-tropic at this time. Therapy was interrupted from 15 April to 15 August 2014 because the patient traveled to Guinea-Bissau and the viral load on return was 17,575 copies/mL. Treatment with MVC+ RAL+ SQV/r was resumed but genotypic tests performed in December 2014 showed resistance to all classes of ARVs and a viral load of 5630 copies/mL. In July 2015, the patient started DTG + AZT + 3TC, but anemia developed due to AZT, so therapy was changed to DTG + FTC/TDF, with some success since the viral load decreased to 1677 copies/mL on 30 November 2015.

Patients 1 and 7, child and mother, form a pair of transmission. Two isolates of the child, 00PTHDECT and 03PTHDECT, and one isolate of the mother, 03PTHCC20, obtained three years apart, were used in this study.

### 4.4. Virus Stocks and Titration

The sixteen primary isolates described in this study were obtained by co-cultivation with peripheral blood mononuclear cells from seronegative subjects, as described by [67]. Different features of fourteen primary isolates were previously described [18,20,68,69]; two new isolates (15PTHCEC and 15PTHJSIG) are presented here for the first time, and their origin is described above. The HIV-2ROD lab-adapted strain was obtained by transfection of HEK-293T cells with pROD10 plasmid using jetPrime transfection reagent (Polyplus). Cell culture supernatant was collected 48 h post-transfection, filtered, and stored at −80 °C. The 50% tissue culture infectious dose (TCID_50_) of each isolate was determined in a single-round viral infectivity assay using a luciferase reporter gene assay in TZM-bl cells. Briefly, 10,000 TZM-bl cells were seeded in 96-well tissue culture plates and incubated overnight. The next day, the growth medium was removed and replaced by 200 µL of fresh growth medium supplemented with DEAE-dextran. A total of 100 µL of virus supernatant was added to the first well, from which serial three-fold dilutions were prepared in the next wells. The assay was performed in quadruplets. Cells were incubated with virus for 48 h before quantification of luciferase expression with the Pierce Firefly Luciferase Glow Assay Kit (Thermo Fisher, Waltham, MA, USA) according to the manufacturer’s instructions. Control wells containing only target cells and growth medium were used to measure background luminescence. The TCID_50_ was calculated using the statistical method of Reed and Muench.

### 4.5. Drug Susceptibility Assays

The antiviral activity of RAL, DTG, BIC, and BSS-730A was evaluated using a single-round viral infectivity assay in TZM-bl cells as described previously [70]. Briefly, cells were treated with several fold dilutions of the compounds for 1 h at 37 °C and then infected with 200 TCID_50_ of each virus. After 48 h, luciferase expression was quantified as described above. The cytotoxicity of the compounds was evaluated using control wells in the absence of the virus. At least two independent experiments were performed for each analysis, and each assay was set up in triplicate wells. IC_50_ and IC_90_ fold-changes of isolates from RAL-naïve patients were relative to HIV-2ROD; IC_50_ and IC_90_ fold-changes of isolates from RAL-experienced patients were relative to the mean IC_50_ and IC_90_ of isolates from RAL-naïve patients. The susceptibility of the isolates to the different drugs was classified according to the IC_50_-fold change as follows: sensitive–< 3, low-level resistance–≥3 < 5, intermediate-level resistance–≥5 < 15, and high-level resistance–≥15 [70].

### 4.6. Instantaneous Inhibitory Potential (IIP)

IIP is a drug parameter that incorporates the slope of the dose-response curve (m), the measured IC_50,_ and clinically relevant concentrations of the drug (D), usually Cmax or Cmin [64,65,66]. IIP was calculated as originally described using the equation IIP = log [1+ (D/IC_50_)^m^] [64]. Slope and IC_50_ values used for the IIP calculations are indicated in Supplementary Tables S1 and S4. Cmax and Cmin for the integrase inhibitors were taken from the literature [71,72] and were as follows: RAL, Cmax = 4.497 μM; Cmin = 0.142 μM; DTG, Cmax = 8.315 μM; Cmin = 2.515 μM; BIC, Cmax = 13.046 μM; Cmin = 5.537 μM. To include the Cmax and Cmin of all INIs, IIP calculations were made using a two-fold decreasing range of drug concentrations starting at 50 μM and ending at 0.098 μM. Clinically relevant concentrations of BSS-730A are not yet known; therefore, IIP values of BSS-730A were determined considering Cmax = 50 μM, Cmed = 3.125 μM, and Cmin = 0.098 μM.

### 4.7. Drug Combination Assays

The combination of BSS-730A and RAL was examined in a single-round viral infectivity assay using TZM-bl reporter cells and 200 TCID_50_ of HIV-2 primary isolate 03PTHCC19. Serial two-fold dilutions of a fixed combination of BSS-730A and RAL were tested. Each concentration of BSS-730A and RAL was also tested alone. Duplicate cultures were maintained for each compound concentration and for infected and uninfected controls. After 48 h of infection, luciferase expression was quantified. The type of interaction was determined by using CompuSyn software (ComboSyn, Inc., Paramus, NJ, USA). Combination indices (CIs) were calculated based on the median-effect principle [73,74], where CI < 0.9 indicates a synergistic effect (CI values were interpreted as follows: 0.9 > CI > 0.85: slight synergism, 0.85 > CI > 0.7: moderate synergism, 0.7 > CI > 0.3: synergism, 0.3 > CI > 0.1: strong synergism, CI < 0.1: very strong synergism), 0.9 < CI < 1.1 indicates an additive effect, and CI >1.1 indicates an antagonistic effect. Because high effect degrees are more important to the treatment than low effect degrees, the weighted average CI value was assigned as CI_wt_ = [CI_50_ + 2 CI_75_ + 3 CI_90_ + 4 IC_95_]/10, where CI_50_, CI_75_ CI_90,_ and CI_95_ are the CI values at 50, 75, 90, and 95% inhibition, respectively [73,74].

### 4.8. DNA Extraction, PCR Amplification and Sequencing

Viral RNA was extracted from 1 mL of cell culture supernatant diluted, according to Biomérieux’s easyMAG automatic extraction procedure. RNA was reverse-transcribed using Qiagen One-Step RT-PCR Kit. To amplify and sequence the integrase (293 amino acids) gene, a nested PCR was performed using the Thermo Scientific Taq DNA Polimerase (recombinant) reagent (Supplementary Table S6). Amplification products were checked on a 1% agarose gel and were subsequently purified using the ExoSAP-IT protocol. The sequencing reaction was performed using the Big Dye Terminator v3.1 Cycle Sequencing Kit (Applied Biosystems). Products were purified and run on an ABI PRISM^®^ 3130 Genetic Analyzer (Applied Biosystems, Waltham, MA, USA). Nucleotide sequences were aligned against the HIV-2 ROD reference strain (GenBank accession # M15390) and edited with SeqScape and ChromasPro Software. Sequences were submitted to GenBank and were given the following accession numbers: KY962712-KY962727.

### 4.9. Phylogenetic Analysis

The nucleotide sequences of the integrase gene were aligned with reference sequences from all HIV-2 groups recovered from Los Alamos HIV Sequence Database (https://www.hiv.lanl.gov/content/sequence/HIV/mainpage.html, accessed on 1 July 2022), using the Muscle program [75] implemented in SeaView version 4.5.4 [76]. Maximum likelihood analysis was performed using the best-fit model of molecular evolution estimated by Find Model (http://www.hiv.lanl.gov/content/sequence/findmodel/findmodel.html accessed on 1 July 2022) under the Akaike Information Criterion. The model chosen was GTR + G. The phylogenetic tree was reconstructed using the PhyML program implemented in SeaView version 4.5.4 [76] using the nearest-neighbor interchange (NNI) heuristic search strategy and 1000 bootstrap replications.

### 4.10. Analysis of Genotypic Drug Resistance

The nucleotide sequence of the IN gene of each HIV-2 primary isolate was analyzed using Genotypic Resistance-Algorithm Deutschland (GRADE) (http://www.hiv-grade.de/HIV2EU/deployed/grade.pl?program=hivalg, accessed on 1 July 2022) and HIVdb Program for HIV-2 (https://hivdb.stanford.edu/ accessed on 1 July 2022) to identify any mutations associated to the resistance of integrase inhibitors and the corresponding level of resistance determined by each mutation. The integrase sequences derived from HIV-2 primary isolates were also compared to the sequence of HIV-2ROD to identify the presence of polymorphisms. The level of resistance for each polymorphism was determined based on the GRADE Algorithm, drug susceptibility assays (fold-change), and what is described in the literature.

### 4.11. Statistical Analysis

Statistical analyses were performed using Prism version 9.2 for Microsoft (GraphPad Software, San Diego, CA, USA, www.graphpad.com accessed on 1 July 2022) with a level of significance of 5%. The 50% inhibitory concentration (IC_50_), 90% inhibitory concentration (IC_90_), and slope (Hill slope) best-fit values were inferred from sigmoidal dose-response (variable slope) curves adjusted to combined results of isolates from RAL-naïve and RAL-experienced patients. One-way ANOVA and the F-test were used to compare best fit-values and fold-change values from drug-naïve or RAL-experienced isolates; Tukey’s multiple comparisons test was used to compare mean values between groups. The Man–Whitney U test was used to compare best-fit values between isolates from drug naïve and RAL-experienced isolates. Despite the same origin of some of our isolates, for statistical analysis, all isolates were considered independent entities because the rate of virus replacement in each patient is very fast due to the high within-patient evolution rate of HIV-2 [57]. This can be seen in phylogenetic analysis, where the genetic distance between some unrelated reference isolates is similar to the genetic distance between the isolates of our patients (Supplementary Figure S2).

## 5. Conclusions

In summary, RAL, DTG, BIC, and BSS-730A show potent in vitro activity against primary HIV-2 isolates from INI-naïve patients. E92A and Q148K confer high-level resistance to RAL, and E92Q and T97A confer resistance to RAL and intermediate-level resistance to DTG. BIC and BSS-730A antiviral activity are not affected by these mutations and should be useful to treat infections caused by RAL-resistant strains. The high IIP of BIC and, especially, of BSS-730A highlights their potential value for treating HIV-2 infection. Overall, the results are consistent with the high clinical efficacy of RAL and DTG for HIV-2 infection and suggest a promising clinical profile for BIC and BSS-730A.

## Figures and Tables

**Table 1:** Comparison of the antiviral activity of the different drugs against isolates from RAL-naïve and RAL-experienced patients.

Parameter ^a^	RAL-Naive Isolates [Mean Values (95% CI)] (*n* = 14)	RAL-Experienced Isolates [Mean Values (95% CI)] (*n* = 3)	*p*-Value ^b^
RALTEGRAVIR			
IC_50_ (nM)	2.692 (1.351, 4.032)	78.178 (−195.1, 351.5)	0.0294
Fold-change IC_50_	1.681 (0.803, 2.559)	29.043 (−72.491, 130.578)	0.1393
IC_90_ (nM)	19.998 (7.463, 32.533)	236.169 (−242.727, 715,065)	0.0324
Fold-change IC_90_	1.066 (0.401, 1.731)	11.809 (−12.138, 35.757)	0.0393
Hill slope	1.44 (0.922, 1.952)	1.95 (−1.952, 5.846)	0.9529
DOLUTEGRAVIR			
IC_50_ (nM)	2.778 (2.037, 3.518)	14.467 (−24.96, 53.89)	0.0059
Fold-change IC_50_	0.970 (0.688, 1.253)	5.208 (−8.985, 19.401)	0.0071
IC_90_ (nM)	16.264 (11.307, 21.221)	89.460 (−70.462, 249,383)	0.0132
Fold-change IC_90_	1.607 (1.099, 2.116)	5.501 (−4.332, 15.334)	0.1464
Hill slope	1.64 (0.925, 2.352)	1.22 (0.276, 2.162)	0.6765
BICTEGRAVIR			
IC_50_ (nM)	2.595 (1.707, 3.483)	8.322 (−14.37, 31.01)	0.3618
Fold-change IC_50_	0.735 (0.458, 1.013)	3.207 (−5.537, 11.951)	0.3536
IC_90_ (nM)	30.493 (17.248, 43.738)	156.100 (−312.135, 624.335)	0.1485
Fold-change IC_90_	2.375 (1.322, 3.427)	5.119 (−10.236, 20.474)	0.7821
Hill slope	1.03 (0.840, 1.228)	1.14 (−0.488, 2.773)	0.5912
BSS-730A			
IC_50_ (nM)	18.101 (13.37, 22.84)	15.667 (7.61, 23.65)	0.6044
Fold-change IC_50_	0.993 (0.710, 1.277)	0.865 (0.425, 1.306)	0.8161
IC_90_ (nM)	46.549 (31.850, 61.247)	42.974 (−0.975, 86.924)	0.9529
Fold-change IC_90_	0.957 (0.607, 1.308)	0.903 (−0.021, 1.827)	>0.9999
Hill slope	2.87 (2.087, 3.651)	2.58 (0.581, 4.579)	>0.9999

^a^ The 50% inhibitory concentration (IC50), 90% inhibitory concentration (IC90), and slope (Hill slope) best-fit values were inferred from sigmoidal dose-response (variable slope) curves adjusted to combined results of isolates from RAL-naïve and RAL-experienced patients. ^b^ *p*-value for comparison of best-fit values using the Mann–Whitney test. Fold-change of IC_50_ and IC_90_ against isolates from RAL-naïve patients is relative to the reference isolate HIV-2ROD; fold-change of IC_50_ and IC_90_ against isolates from RAL-experienced patients is relative to isolates from RAL-naïve patients.

**Table 2:** Combination indices (CI) of different RAL: BSS-730A combinations against HIV-2.

Drug Combination (Combination Ratio)	CI Values at Inhibition of ^1^:				CI_wt_-Values ^3^
	50%	75%	90%	95%	
RAL + BSS-730A (1:1)	0.24265	0.17127	0.18068	0.19165	0.19
	++++ ^2^	++++	++++	++++	++++
RAL + BSS-730A (1:3)	0.17775	0.16790	0.18613	0.20118	0.15
	++++	++++	++++	++++	++++
RAL + BSS-730A (3:1)	0.09422	0.05240	0.06646	0.08334	0.07
	+++++	+++++	+++++	+++++	+++++

^1^ CI > 1.1 indicates antagonism (−), 1.1 > CI > 0.9 indicates the additive effect (ad) and CI < 0.9 indicates a synergistic effect; ^2^ Synergy levels: 0.9 > CI > 0.85: + (slight synergism); 0.85 > CI > 0.7: ++ (moderate synergism); 0.7 > CI > 0.3: +++ (synergism); 0.3 > CI > 0.1: ++++ (strong synergism); CI < 0.1; +++++ (very strong synergism); ^3^ Weighted average CI value determined as follows: CI_wt_ = [CI_50_ + 2 CI_75_ + 3 CI_90_ + 4 CI_95_]/10.

**Table 3:** Resistance mutations in the integrase protein of RAL-experienced patients and susceptibility to RAL and DTG.

Patient (Isolate)	Major Resistance Mutations	Accessory Resistance Mutations	Predicted Susceptibility *		
			RAL	DTG	BIC **
10PTHSJIG	E92Q	T97A	Resistant	Intermediate	Sensitive
15PTHSJIG	None	I84V	Sensitive	Sensitive	Sensitive
15PTHCEC	E92A, Q148K	I84V	Resistant	Resistant	Sensitive

* Based on the GRADE algorithm and ** current literature.

## Data Availability

Sequences analyzed in this paper are available in GenBank using accession numbers: KY962712-KY962727.

## Supplementary Materials

The following supporting information can be downloaded at: https://www.mdpi.com/article/10.3390/ijms232214300/s1, Figure S1: Evolution of viral load and CD4+ T cell counts in patients on a DTG-based treatment regimen after failing a RAL-based regimen. (A) Patient 10; (B) Patient 14. The arrow indicates the approximate time of virus isolation. R5 corresponds to CCR5 coreceptor use and X4 to CXCR4 coreceptor use as determined by genotypic analysis of the sequence of the V3 region of the isolates. The gray rectangles indicate periods of drug interruption. Combivir [lamivudine (3TC) and zidovudine (AZT)]; DRVr, ritonavir boosted darunavir; SQVr, ritonavir boosted saquinavir; FTC, emtricitabine; TDF, tenofovir disoproxil fumarate; MVC, maraviroc; RAL, raltegravir; DTG, dolutegravir. Figure S2: Phylogenetic tree of the integrase genes from HIV-2 primary isolates. The maximum likelihood phylogenetic tree was constructed with reference sequences from all HIV-2 subtypes. The bootstrap values supporting the internal branches are shown. Only bootstrap values above 70% are shown. The red box indicates the cluster containing the sequences of isolates from Patients 1 (00PTHDECT and 03 PTHDECT) and 7 (03PTHCC20), son and mother, respectively, which form a pair of transmission. Table S1: Characterization of the HIV-2 infected patients from which the viruses were isolated. Table S2: Hill slope values for all drugs and isolates. Table S3: Inhibitory concentration 50% (IC_50_) and IC_50_ fold-change of RAL, DTG, BIC, and BSS-730A against primary HIV-2 isolates from RAL-naïve and RAL-experienced patients. Table S4: PCR integrase primers. Table S5: Inhibitory concentration 90% (IC_90_) and IC_90_ fold-change of RAL, DTG, BIC, and BSS-730A against primary HIV-2 isolates from RAL-naïve and RAL-experienced patients. Table S6: Instantaneous Inhibitory Potential (IIP) values for all isolates and drugs.
