# Supplementary material for: High Instantaneous Inhibitory Potential of Bictegravir and the New Spiro-β-Lactam BSS-730A for HIV-2 Isolates from RAL-Naïve and RAL-Failing Patients
Source: Int J Mol Sci. 2022 Nov 18;23(22):14300. doi: 10.3390/ijms232214300 (PMC9695772; doi:10.3390/ijms232214300)
Supplement: Supplementary file 1 [file ijms-23-14300-s001.zip › ijms-1984255-supplementary.pdf]

**Supplementary Figures**

**A)**

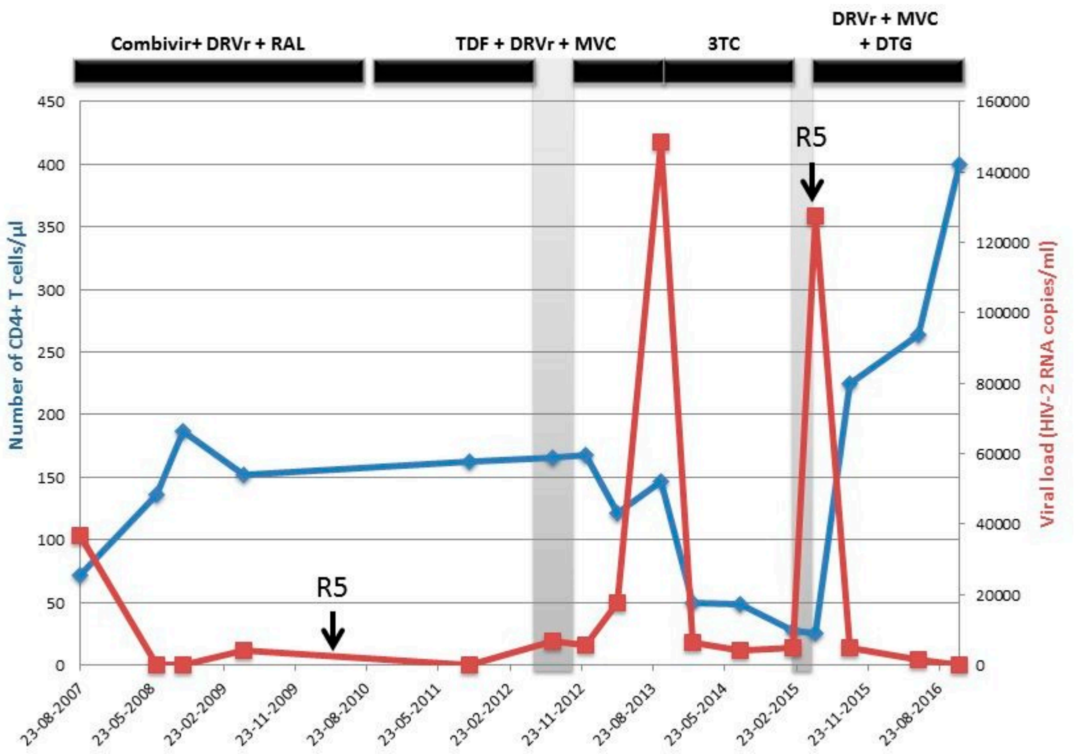


**B)**

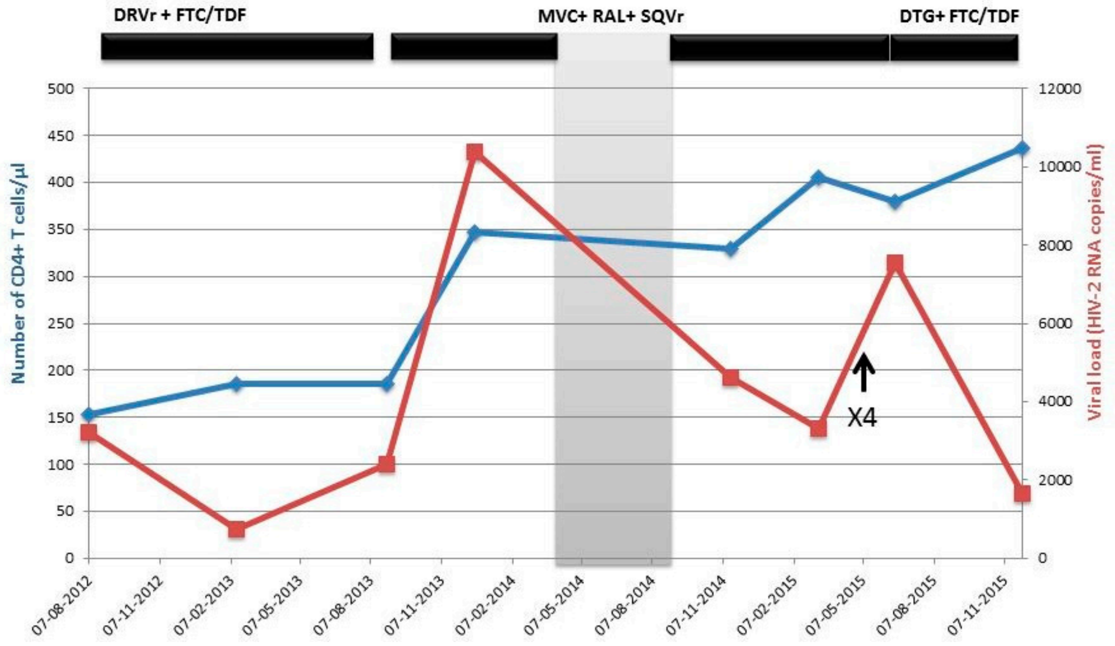

**Figure S1**: Evolution of viral load and CD4+ T cell counts in patients on a DTG- based treatment regimen after failing a RAL-based regimen. A) Patient 10; B) Patient 14. The arrow indicates the approximate time of virus isolation. R5 corresponds to CCR5 coreceptor use and X4 to CXCR4 coreceptor use as determined by genotypic analysis of the sequence of the V3 region of the isolates. The gray rectangles indicate periods of drug interruption. Combivir [lamivudine (3TC) and zidovudine (AZT)]; DRVr, ritonavir boosted darunavir; SQVr, ritonavir boosted saquinavir; FTC, emtricitabine; TDF, tenofovir disoproxil fumarate; MVC, maraviroc; RAL, raltegravir; DTG, dolutegravir.

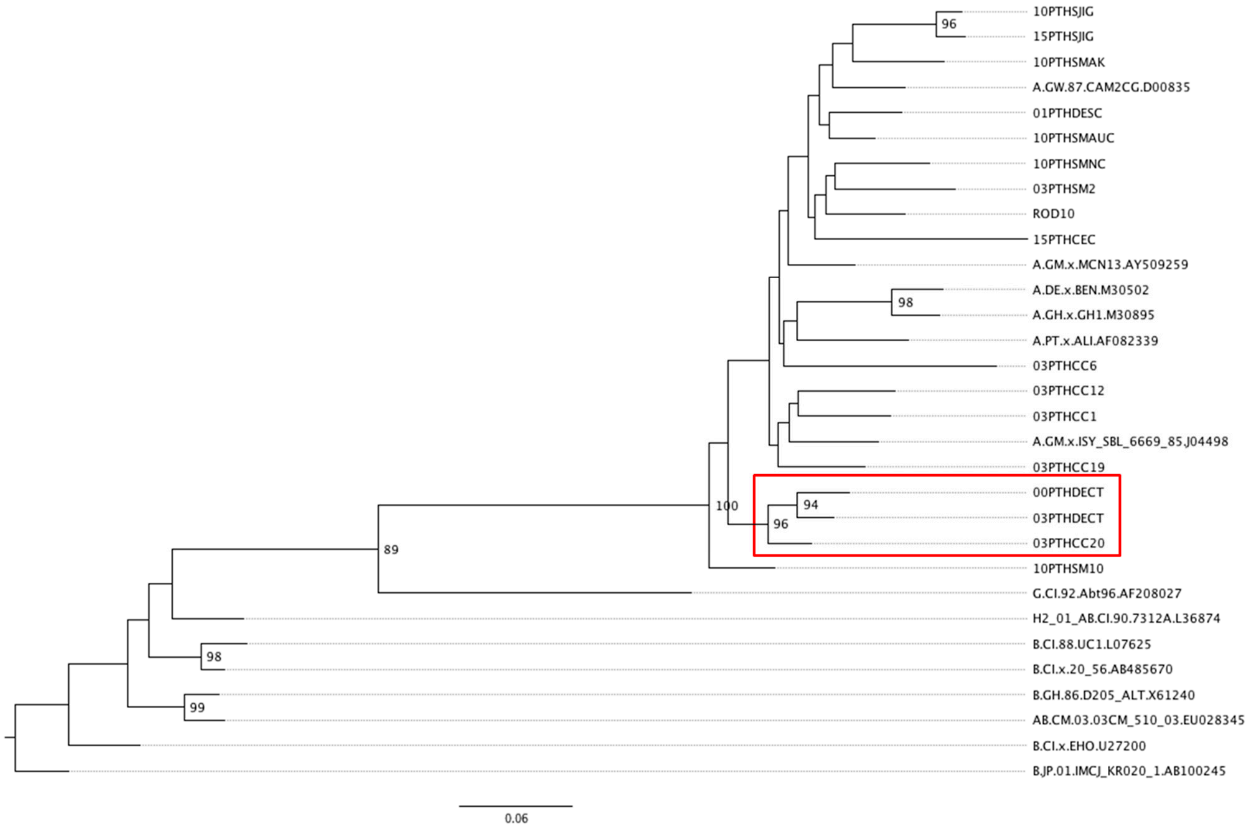


**Figure S2:** Phylogenetic tree of the integrase genes from HIV-2 primary isolates. The maximum likelihood phylogenetic tree was constructed with reference sequences from all HIV-2 subtypes. The bootstrap values supporting the internal branches are shown. Only bootstrap values above 70% are shown. The red box indicates the cluster containing the sequences of isolates from patients 1 (00PTHDECT and 03 PTHDECT) and 7 (03PTHCC20), son and mother, respectively, which form a pair of transmission.

**Supplementary Tables**

**Table S1**: Inhibitory concentration 50% ($IC_{50}$) and $IC_{50}$ fold-change of RAL, DTG, BIC and BSS-730A against primary HIV-2 isolates from RAL-naïve and RAL-experienced patients.

| Type of patient | Name of virus isolates | RAL | | DTG | | BIC | | BSS-730A | |
|---|---|---|---|---|---|---|---|---|---|
| | | Mean values (95% CI) (nM) | Fold change* | Mean values (95% CI) (nM) | Fold change* | Mean values (95% CI) (nM) | Fold change* | Mean values (95% CI) (nM) | Fold change* |
| RAL – naïve patients | ROD10 | ~1.649 (Very wide) | - | 2.856 (1.965-3.877) | - | 3.440 (2.335-5.066) | - | 18.210 (14.38-23.05) | - |
| | 00PTHDECT | 4.127 (3.132-4.718) | 2.503 | 2.765 (1.267-5.246) | 0.968 | 2.075 (1.239-3.367) | 0.603 | 23.000 (16.50-22.96) | 1.263 |
| | 03PTHDECT | 7.502 (5.689-8.583) | 4.549 | 3.873 (2.982-5.747) | 1.356 | 6.971 (5.342-8.082) | 2.026 | 30.000 (23.79-32.08) | 1.647 |
| | 03PTHCC20 | 2.583 (2.134-2.468) | 1.566 | ~2.478 (Very wide) | 0.868 | 1.879 (1.297-2.742) | 0.546 | 18.000 (12.96-23.94) | 0.988 |
| | 01PTHDESC | 3.334 (1.916-6.069) | 2.022 | 1.035 (0.629-1.627) | 0.362 | 2.778 (1.900-3.209) | 0.808 | 4.965 (42.40-58.32) | 0.273 |
| | 03PTHCC1 | 6.336 (5.933-6.767) | 3.842 | 1.395 (0.915-2.326) | 0.488 | 1.768 (1.361-2.253) | 0.514 | 13.400 (13.11-13.70) | 0.736 |
| | 03PTHCC6 | 4.550 (3.953-5.242) | 2.759 | 5.612 (3.254-9.449) | 1.965 | 2.432 (2.144-2.727) | 0.707 | 25.190 (12.49-50.80) | 1.383 |
| | 03PTHCC12 | 0.1400 (0.07-0.2567) | 0.085 | 2.088 (1.673-2.294) | 0.731 | 1.769 (1.474-2.308) | 0.514 | 14.220 (13.88-14.62) | 0.781 |
| | 03PTHCC19 | 2.775 (1.827-3.657) | 1.683 | 1.052 (0.882-1.378) | 0.368 | 1.955 (1.521-2581) | 0.568 | 8.000 (5.795-11-02) | 0.439 |
| | 03PTHSM2 | 0.1900 (0.083-0.435) | 0.115 | 3.442 (2.236-5.458) | 1.205 | 1.783 (1.350-2.083) | 0.518 | 12.080 (11.70-12.52) | 0.663 |
| | 04PTHSM10 | 1.611 (1.262-2.058) | 0.977 | 2.093 (1.748-2.316) | 0.733 | 0.920 (0.600-1.168) | 0.267 | 12.890 (12.49-13.37) | 0.708 |
| | 10PTHSMAK | 0.1400 (0.078-0.254) | 0.085 | 4.327 (3.654-4.971) | 1.515 | 3.325 (2.794-3778) | 0.967 | 29.610 (21.20-31.79) | 1.626 |
| | 10PTHSMAUC | 2.531 (2.148-2.984) | 1.535 | 2.557 (2.388-2.712) | 0.895 | 4.076 (3.611-4.705) | 1.185 | 14.120 (13.74-14.56) | 0.775 |

| | | | | | | | | | |
|---|---|---|---|---|---|---|---|---|---|
| | 10PTHSMNC | 0.217 (0.07-0.67) | 0.132 | 3.317 (2.469-4.552) | 1.161 | 1.160 (0.618-2.070) | 0.337 | 29.73 (13.19-66.98) | 1.633 |
| **RAL - experienced patients** | 10PTHSJIG | 25.830 (1.610-3.596) | 9.596 | 32.790 (28.60-37.74) | 11.804 | 18.630 (15.35-20.71) | 7.179 | 12.000 (10.95-13.52) | 0.663 |
| | 15PTHSJIG | 4.105 (3.408-5.033) | 1.525 | 4.967 (4.289-5.671) | 1.788 | 5.107 (3.741-6.339) | 1.968 | 18.000 (16.95-24.17) | 0.994 |
| | 15PTHCEC | 204.600 (125.3-334.0) | 76.009 | 5.644 (4.242-7.559) | 2.032 | 1.229 (0.616-2.434) | 0.474 | 17.000 (15.53-19.45) | 0.939 |

*$IC_{50}$ fold-change of isolates from RAL-naïve patients was relative to HIV-2 ROD. $IC_{50}$ fold-change of isolates from RAL-experienced patients was relative to those from naïve patients. CI - Confidence Intervals

**Table S2**: Inhibitory concentration 90% ($IC_{90}$) and $IC_{90}$ fold-change of RAL, DTG, BIC and BSS-730A against primary HIV-2 isolates from RAL-naïve and RAL-experienced patients.

| Type of patient | Name of virus isolates | RAL | | DTG | | BIC | | BSS-730A | |
|---|---|---|---|---|---|---|---|---|---|
| | | Mean values (95% CI) (nM) | Fold change* | Mean values (95% CI) (nM) | Fold change* | Mean values (95% CI) (nM) | Fold change* | Mean values (95% CI) (nM) | Fold change* |
| RAL – naïve patients | ROD10 | ~2.800 (Very wide) | - | 10.423 (1.8-59.9) | - | 13.397 (4.159-43.25) | - | 49.545 (12.8-191.6) | - |
| | 00PTHDECT | 22.080 (14.2-34.4) | 1.104 | 19.364 (5.4-68.9) | 1.858 | 40.700 (17.8-93.1) | 3.038 | 57.411 (41.9-78.9) | 1.159 |
| | 03PTHDECT | 76.913 (52.2-113.1) | 3.846 | 22.751 (13.6-37.8) | 2.183 | 56.600 (37.7-84.9) | 4.225 | 81.846 (64.4-104.2) | 1.652 |
| | 03PTHCC20 | 9.750 (7.3-13.0) | 0.488 | ~3.936 (Very wide) | 0.377 | 67.800 (29.6-155.5) | 5.061 | 41.210 (17.7-95.9) | 0.832 |
| | 01PTHDESC | 39.626 (14.9-105.0) | 1.981 | 35.075 (16.7-73.6) | 3.365 | 21.300 (11.6-39.1) | 1.590 | 15.812 (14.5-17.3) | 0.319 |
| | 03PTHCC1 | 30.974 (27.7-34.7) | 1.549 | 19.409 (6.9-54.6) | 1.862 | 9.900 (5.0-19.3) | 0.739 | 22.594 (22.16-23.7) | 0.456 |
| | 03PTHCC6 | 17.179 (12.8-23.1) | 0.859 | 17.906 (6.6-48.6) | 1.769 | 10.800 (6.8-17.0) | 0.806 | 61.376 (9.4-399.7) | 1.239 |
| | 03PTHCC12 | 0.603 (0.20-1.70) | 0.030 | 7.816 (4.6-13.3) | 0.750 | 12.600 (7.2-22.1) | 0.941 | 20.654 (19.0-22.4) | 0.417 |
| | 03PTHCC19 | 44.463 (20.3-97.3) | 2.223 | 8.279 (4.5-15.3) | 0.794 | 31.800 (21.5-46.9) | 2.374 | 74.300 (41.6-132.7) | 1.500 |
| | 03PTHSM2 | 0.986 (0.3-3.0) | 0.049 | 22.029 (5.8-83.9) | 2.113 | 7.000 (3.9-12.5) | 0.523 | 18.923 (18.2-19.7) | 0.382 |
| | 04PTHSM10 | 15.031 (8.4-27.0) | 0.752 | 9.977 (6.5-15.3) | 0.957 | 16.100 (8.3-31.4) | 1.202 | 24.831 (23.2-26.7) | 0.501 |
| | 10PTHSMAK | 4.450 (1.6-12.7) | 0.222 | 18.793 (12.8-27.6) | 1.803 | 21.300 (15.1-30.0) | 1.590 | 91.20 (65.5-170.3) | 2.132 |
| | 10PTHSMAUC | 12.910 (6.7-24.8) | 0.646 | 7.620 (4.4-13.2) | 0.731 | 40.000 (29.6-54.0) | 2.986 | 28.445 (26.3-30.8) | 0.574 |
| | 10PTHSMNC | 2.213 (0.50-9.70) | 0.111 | 24.322 (10.5-56.1) | 2.333 | 77.6 (21.4-281.2) | 5.794 | 63.533 (11.7-344.3) | 1.282 |

| | | | | | | | | | |
|---|---|---|---|---|---|---|---|---|---|
| **RAL - experienced patients** | 10PTHSJIG | 275.93 (98.6-772.9) | 13.798 | 151.22 (92.3-247.9) | 9.298 | 76.700 (49.5-118.9) | 2.515 | 22.751 (3.1-166.1) | 0.478 |
| | 15PTHSJIG | 26.607 (17.5-40.3) | 1.330 | 22.751 (16.6-31.1) | 1.399 | 20.300 (11.7-35.3) | 0.666 | 55.590 (40.9-75.5) | 1.168 |
| | 15PTHCEC | 405.97 (0.1-1733) | 20.300 | 94.410 (51.9-171.7) | 5.805 | 371.300 (114.7-1202) | 12.176 | 50.582 (37.5-58.2) | 1.063 |

*$IC_{90}$ fold-change of isolates from RAL-naïve patients was relative to HIV-2 ROD. $IC_{90}$ fold-change of isolates from RAL-experienced patients was relative to those from naïve patients. CI - Confidence Intervals.

**Table S3:** Characterization of the HIV-2 infected patients from which the viruses were isolated.

| Patient number | Isolate ID | Date of sample collection | Year of diagnosis | Gender | CD4+ T cell count/ul | RNA copies/ ml | Date of starting therapy | ARTc | Co-receptor use |
|---|---|---|---|---|---|---|---|---|---|
| 1 | 00PTHDECT | 2000 | 1998 | M | 2919 | na | Untreated | - | R5/X4 |
| | 03PTHDECT | 2003 | 1998 | M | 209 | 20968 | 2003 | d4T,3TC, LPV/r | X4 |
| 2 | 01PTHDESC | 2001 | 1992 | F | 44 | 1250 | na | AZT, 3TC | X4 |
| 3 | 03PTHCC1 | 2003 | 2001 | F | 308 | <200 | 2001 | DDI, D4T, IDV | R5 |
| 4 | 03PTHCC6 | 2003 | 1992 | F | 615 | <200 | 1996 | AZT,3TC, IDV | R5 |
| 5 | 03PTHCC12 | 2003 | 1995 | M | 66 | <200 | Untreated | - | R5 |
| 6 | 03PTHCC19 | 2003 | 2003 | F | 175 | <200 | 2005 | D4T, 3TC, LPV/r | R5 |
| 7 | 03PTHCC20 | 2003 | 1998 | F | 78 | na | 2005 | TDF, ABC, LPV/r | X4 |
| 8 | 03PTHSM2 | 2003 | 2002 | M | 275 | <200 | 2002 | AZT, 3TC, DDI | R5 |
| 9 | 04PTHSM10 | 2004 | 2001 | F | 265 | 4792 | 2002 | AZT, 3TC, NVF | X4 |
| 10 | 10PTHSJIG | 2010 | 2005 | F | 164 | 4257 | na | AZT, 3TC, RAL | R5 |
| | 15PTHSJIG | 2015 | 2005 | F | 40 | 1793 | na | DRV/r, MVC, DTG | R5 |
| 11 | 10PTHSMAK | 2010 | 2009 | F | 40 | 1793 | Untreated | - | X4 |
| 12 | 10PTHSMAUC | 2010 | 2010 | M | 177 | <200 | Untreated | - | X4 |
| 13 | 10PTHSMNC | 2010 | 2008 | F | 231 | <200 | na | SQV, ABC, 3TC | R5/X4 |
| 14 | 15PTHCEC | 2015 | 2010 | F | 436 | 1677 | 2010 | MVC, RAL, SQV/r | X4 |

**Table S4**: Hill slope values for all drugs and isolates.

| Isolates | Hill slope values | | | |
|---|---|---|---|---|
| | **RAL** | **DTG** | **BIC** | **BSS-730A** |
| **RAL-naive patients** | | | | |
| ROD10 | 4.337 | 1.654 | 1.506 | 2.193 |
| 03PTHCC1 | 1.384 | 0.849 | 1.272 | 4.203 |
| 03PTHCC6 | 1.655 | 1.873 | 1.471 | 2.465 |
| 03PTHCC12 | 1.464 | 1.589 | 1.142 | 5.920 |
| 00PTHDECT | 1.257 | 1.090 | 0.734 | 2.031 |
| 03PTHDECT | 0.916 | 1.290 | 1.021 | 2.022 |
| 03PTHCC19 | 0.772 | 1.089 | 0.792 | 0.985 |
| 03PTHCC20 | 1.519 | 5.725 | 0.613 | 2.587 |
| 01PTHDESC | 1.567 | 0.620 | 1.020 | 1.899 |
| 03PTHSM2 | 1.336 | 1.193 | 1.536 | 4.906 |
| 04PTHSM10 | 0.984 | 1.371 | 0.743 | 3.357 |
| 10PTHSMAK | 0.634 | 1.481 | 1.169 | 1.566 |
| 10PTHSMAUC | 1.348 | 2.005 | 0.967 | 3.141 |
| 10PTHSMNC | 0.945 | 1.109 | 0.486 | 2.895 |
| **RAL-experienced patients** | | | | |
| 10PTHSJIG | 0.901 | 1.439 | 1.505 | 3.507 |
| 15PTHSJIG | 1.189 | 1.438 | 1.538 | 2.176 |
| 15PTHCEC | 3.752 | 0.781 | 0.385 | 2.058 |

**Table S5**: Instantaneous Inhibitory Potential (IIP) values for all isolates and drugs.

| Isolates | Instantaneous Inhibitory Potential (IIP) values | | | | | | | |
|---|---|---|---|---|---|---|---|---|
| | RAL | | DTG | | BIC | | BSS-730A | |
| **RAL-naive patients** | **Cmax** | **Cmin** | **Cmax** | **Cmin** | **Cmax** | **Cmin** | **Cmax** | **Cmin** |
| ROD10 | 14.901 | 8.395 | 5.730 | 4.871 | 5.390 | 4.829 | 7.541 | 1.610 |
| 03PTHCC1 | 3.946 | 1.876 | 3.205 | 2.764 | 4.920 | 4.447 | 15.013 | 3.626 |
| 03PTHCC6 | 4.957 | 2.475 | 5.939 | 4.966 | 5.486 | 4.939 | 8.129 | 1.466 |
| 03PTHCC12 | 6.598 | 4.402 | 5.721 | 4.895 | 4.417 | 3.992 | 20.993 | 4.954 |
| 00PTHDECT | 3.818 | 1.937 | 3.791 | 3.225 | 2.789 | 2.517 | 6.778 | 1.298 |
| 03PTHDECT | 2.546 | 1.199 | 4.298 | 3.628 | 3.341 | 2.961 | 6.515 | 1.075 |
| 03PTHCC19 | 2.480 | 1.340 | 4.245 | 3.679 | 3.029 | 2.734 | 3.741 | 1.106 |
| 03PTHCC20 | 4.923 | 2.645 | 20.185 | 17.212 | 2.358 | 2.131 | 8.909 | 1.905 |
| 01PTHDESC | 4.905 | 2.555 | 2.421 | 2.101 | 3.745 | 3.366 | 7.602 | 2.458 |
| 03PTHSM2 | 5.844 | 3.840 | 4.036 | 3.417 | 5.936 | 5.364 | 17.745 | 4.453 |
| 04PTHSM10 | 3.392 | 1.920 | 4.934 | 4.222 | 3.085 | 2.809 | 12.047 | 2.953 |
| 10PTHSMAK | 2.859 | 1.912 | 4.863 | 4.094 | 4.201 | 3.766 | 5.054 | 0.874 |
| 10PTHSMAUC | 4.381 | 2.360 | 7.042 | 6.001 | 3.390 | 3.030 | 11.148 | 2.639 |
| 10PTHSMNC | 4.080 | 2.663 | 3.770 | 3.194 | 1.975 | 1.796 | 9.339 | 1.509 |
| **RAL-experienced patients** | | | | | | | | |
| 10PTHSJIG | 2.022 | 0.752 | 3.460 | 2.713 | 4.282 | 3.722 | 12.695 | 3.193 |
| 15PTHSJIG | 3.614 | 1.837 | 4.636 | 3.889 | 5.240 | 4.668 | 7.493 | 1.609 |
| 15PTHCEC | 5.035 | 0.099 | 2.476 | 2.072 | 1.560 | 1.421 | 7.138 | 1.574 |

**Table S6:** PCR integrase primers.

| Designation | Position* | Sequence |
|---|---|---|
| **PCR primers** | | |
| IN (F) | 3698-3719 | TACACAGATGGATCMTGYAATA |
| POL_inner (R) | 5024-5044 | AATATTACCCTGCTGCAAGTC |
| **Nested primers** | | |
| IN (F) inner | 4343-4364 | AGACTCACARTATGTAATGGG |
| IN (R) inner | 5562-5583 | GCCATCCTTTTTCTGGTGTTA |

*HIV-2_ROD10
